# Dazhu Hongjingtian Injection for Ischemic Stroke: Protocol for a Prospective, Multicenter Observational Study

**DOI:** 10.2196/52447

**Published:** 2023-12-22

**Authors:** Qianzi Che, Tian Song, Ning Liang, Jing Guo, Zhao Chen, Xiaoyu Liu, Lu Yang, Yin Jiang, Yanping Wang, Nannan Shi

**Affiliations:** 1 Institute of Basic Research in Clinical Medicine China Academy of Chinese Medical Sciences Beijing China

**Keywords:** ischemic stroke, study protocol, complementary and alternative therapies, prospective study, stroke, observational study, effectiveness, treatment, optimal treatment, medication, DZHJTI treatment, clinical setting, therapy, cohort study, androgen syndrome, patients with AIS, patient with AIS, China, stroke recurrence, traditional medicine, laboratory parameters, health, tissue plasminogen activator (tPA), tPA, stroke treatment, plasminogen

## Abstract

**Background:**

Although results from in vitro studies and small randomized controlled trials have shown positive effects of Dazhu hongjingtian injection (DZHJTI) on acute ischemic stroke (AIS), their generalizability to routine clinical practice remains to be established.

**Objective:**

The primary aim of this study is to evaluate the effectiveness of DZHJTI treatment for AIS with regard to changes in the stroke-related neurological deficit from baseline to outpatient follow-up, mortality, subsequent vascular events, disability, and traditional Chinese medicine syndrome in real-world clinical settings. By monitoring for adverse events or significant changes in vital signs and laboratory parameters, we also aim to assess the safety of DZHJTI.

**Methods:**

This prospective, multicenter cohort study plans to enroll 2000 patients with AIS within 14 days of symptom onset from 30 hospitals across China. Eligible patients will be followed up for 6 months after initiating medication treatments. The primary outcome will be the change in the National Institute of Health Stroke Scale score from baseline to outpatient follow-up. The secondary outcomes include overall mortality, stroke recurrence, new-onset major vascular events, global disability, and improvement of traditional Chinese medicine syndrome in 6 months. Adverse events or clinically significant changes in vital signs and laboratory parameters, regardless of the severity, will be recorded during the trial to assess the safety of DZHJTI. An augmented inverse propensity weighted estimator will be used to reduce variability and improve accuracy in average treatment effects estimation.

**Results:**

The clinical trial registration was approved in October 2022, and the recruitment and enrollment of participants started in November 2022. The study’s outcomes are expected to be published in 2025 in reputable, peer-reviewed health-related research journals.

**Conclusions:**

This real-world cohort study is the first to assess the effectiveness and safety of DZHJTI in treating AIS. It may provide additional clinical evidence, including the duration of response, long-term drug effectiveness, and subgroup efficacy data. The study results will be valuable for clinicians and patients seeking optimal treatment for AIS and could lead to better use of DZHJTI and improved patient outcomes.

**Trial Registration:**

ITMCTR ITMCTR2022000005; http://tinyurl.com/554ns8m5

**International Registered Report Identifier (IRRID):**

DERR1-10.2196/52447

## Introduction

The high morbidity, mortality, and disability rates associated with stroke impose a significant health and economic burden not only in China but globally [1]. The annual stroke mortality rate in China is approximately 153.9 per 100,000 people, which is 4 times that of American and European countries [1-3]. As the most common subtype of stroke, ischemic strokes accounted for 82.6% (2,818,875/3,411,168) of stroke cases in China admitted in 2019 [4]. Despite receiving guideline-recommended care, including intravenous thrombolysis and endovascular therapy, many patients with stroke continue to experience nerve dysfunction. Recent research conducted in 30 provinces of China revealed that up to 78.7% of first-ever stroke survivors developed poststroke cognitive impairment within 3-6 months following the initial stroke event [5]. This highlights the urgent need to identify a medication with proven efficiency and safety in improving neurofunctional recovery, thereby alleviating the substantial burden on patients and the health care system.

**Figure 1:**
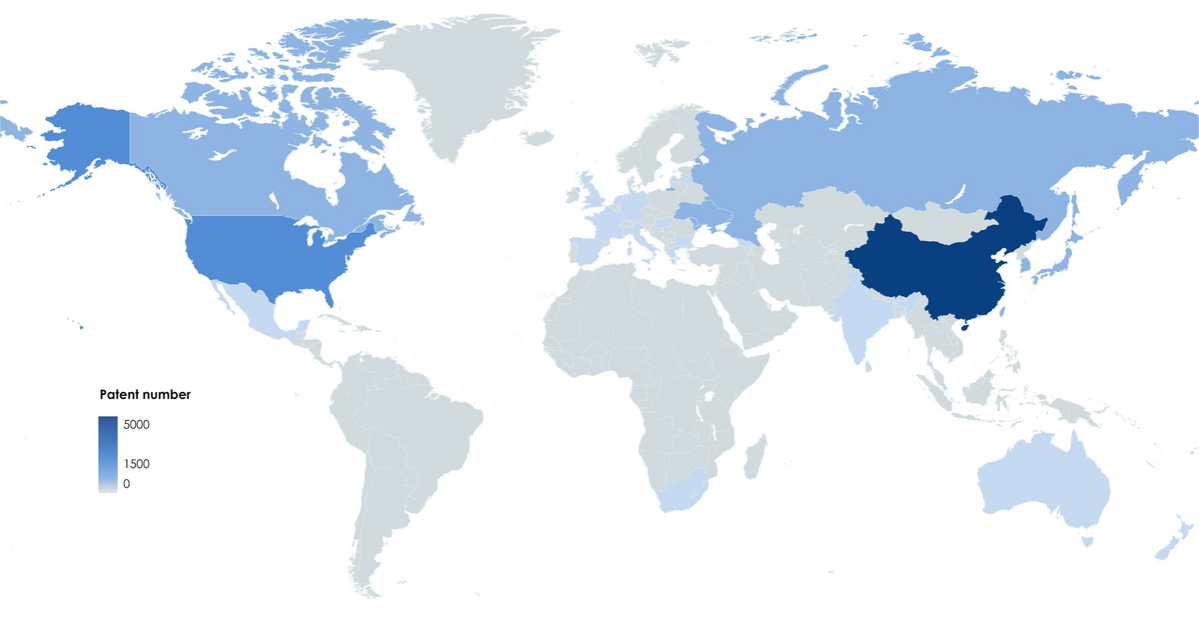
Worldwide patent application for Rhodiola. Data collected from the Lens (searching term: Rhodiola) as of July 20, 2023.

Dazhu hongjingtian (DZHJT) or *Rhodiola wallichiana* var *cholaensis* (Praeger) SH Fu belongs to the family Crassulaceae in the genus *Rhodiola*. Plants of this genus have a long history of traditional use and are included in the official pharmacopeia of Asia, Europe, North America, Latin America, Oceania, and Africa, which is attributed to their proven efficacy in stimulating the nervous system, alleviating fatigue, and preventing high altitude sickness [6,7] (Figure 1). Rhodiola dietary supplements have been recognized by the European Food Safety Authority for their contribution to optimal mental and cognitive activity [8]. DZHJT injection (DZHJTI) is produced from dried roots and stems of DZHJT using processes approved by the Food and Drug Administration of China. Extensive research on the chemical constituents of DZHJTI has identified a total of 49 compounds using ultra-high–performance liquid chromatography coupled with quadrupole time-of-flight mass spectrometry analysis, and a fingerprint has been established by high performance liquid chromatography [9,10]. The primary active constituent of DZHJT, salidroside, has demonstrated various biological activities in both in vitro and in vivo studies, including effects on the central nervous system, cardiac vessel dilation, antihypoxia, and anti-inflammatory properties, among others [11-13].

Several clinical studies have investigated the effects of DZHJTI on acute ischemic stroke (AIS). A pooled analysis of 18 trials involving 1713 participants demonstrated that DZHJTI combined with guideline-recommended therapy was superior to the control group in decreasing the National Institute of Health Stroke Scale (NIHSS; standardized mean difference –0.77, 95% CI –1.52 to –0.02). Moreover, when combined with basic therapy, DZHJTI can also significantly improve the whole blood viscosity, plasma viscosity, and level of fibrinogen, as well as decrease fasting blood glucose and levels of total cholesterol [14]. However, the relatively insufficient sample sizes and ambiguous study designs of previous studies urge high-quality real-world evidence from a rigorous research process.

To enhance the quality of clinical evidence and promote standardized development in clinical research related to traditional medicine on a global scale, we have established the International Traditional Medicine Clinical Trial Registry (ITMCTR), the world’s first international clinical trial registration platform for traditional medicine. This accomplishment has earned ITMCTR the distinction of being approved as a level 1 World Health Organization international clinical trial registration institution. ITMCTR became a member of the Primary Registry Network of the International Clinical Trials Registry Platform and the trials registered with ITMCTR have been added to the International Clinical Trials Registry Platform database. Building upon the foundation of this endeavor, we conducted a multicenter, prospective cohort study to assess the effectiveness and safety of DZHJTI as adjuvant therapy for patients with AIS in a real-world setting. With a complete and accurate study design and prospective registration, this study will provide qualified evidence for DZHJTI.

## Methods

### Study Design

**Figure 2:**
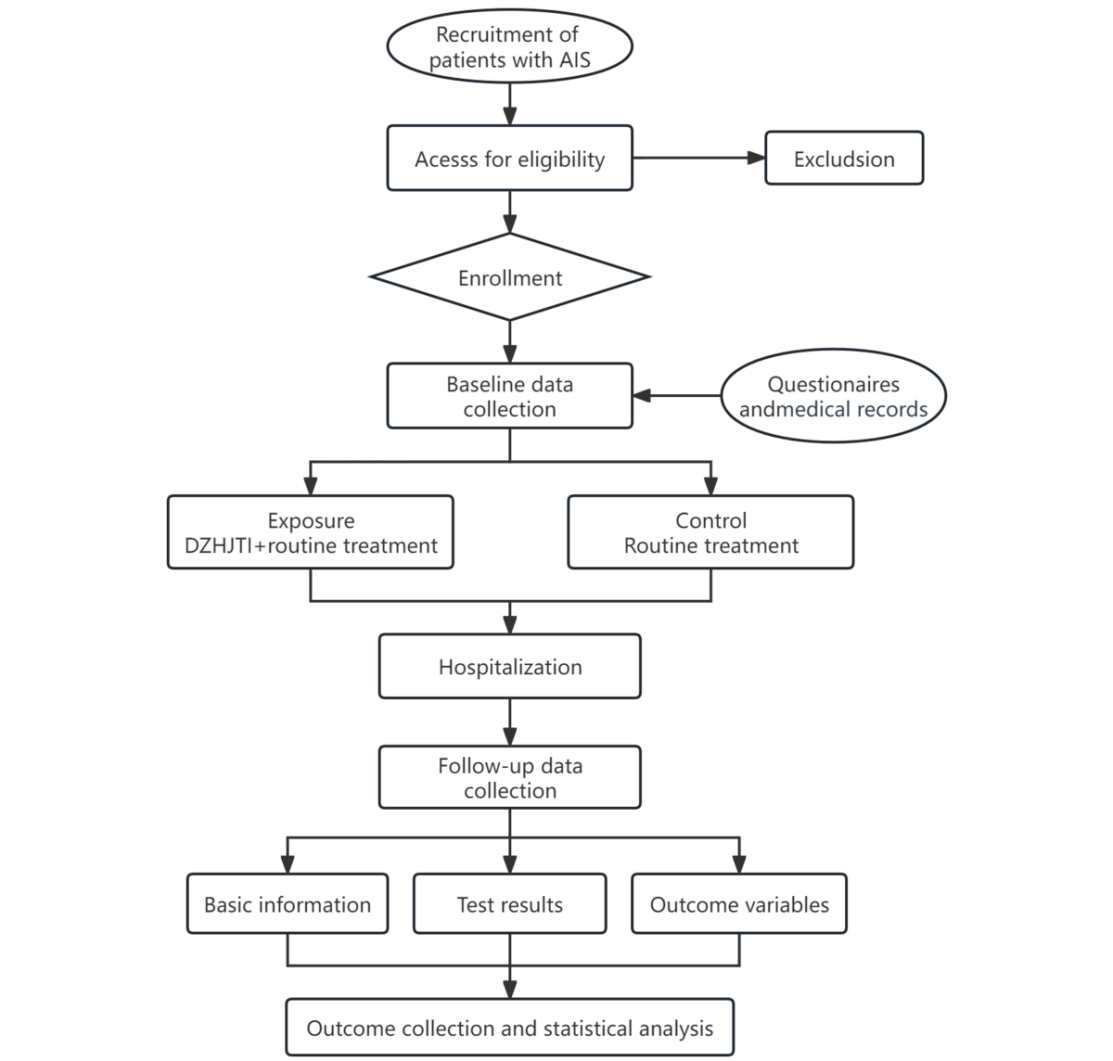
Flowchart of the study design. AIS: acute ischemic stroke; DZHJTI: Dazhu hongjingtian injection.

Our study is a prospective, multicenter cohort study conducted in a real-world setting. An overview of the study flowchart is presented in Figure 2. It is planned to enroll 2000 patients with AIS, as defined in the *Chinese Guidelines for Diagnosis and Treatment of Acute Ischemic Stroke 2018* [15] and *Stroke Diagnosis and Effectiveness Evaluation Standards* [16]. A total of 3 visits will be carried out within a 6-month follow-up period. Baseline demographic data, therapeutic effects, adverse events, and complications associated with DZHJTI will be analyzed.

### Participants

To collect a representative sample, the nephrology departments of 30 secondary or tertiary hospitals in multiple regions of China will continuously recruit and enroll patients. Recruitment began in November 2022 and will continue until at least 2000 participants are recruited. Potential patients will undergo eligibility screening based on the inclusion and exclusion criteria. Eligible patients or their legally authorized representatives will provide written informed consent before undergoing any study-specific procedures. Textbox 1 provides detailed information on the inclusion and exclusion criteria.

**Inclusion and exclusion criteria of the cohort study.**

**Inclusion criteria**

- Female or male patients aged ≥18 years
- Meeting the acute ischemic stroke diagnostic criteria
- Time from symptom onset to the hospital admission ≤14 days
- Willingly participates in the clinical study and signs the informed consent

**Exclusion criteria**

- Clinically silent acute ischemic stroke
- Minor stroke or minor neurological deficit (National Institute of Health Stroke Scale score of 0-5), nondisabling or rapidly improving transient cerebral ischemia
- Severe hypertension or diabetes that is still unable to control the disease after treatment
- Other conditions that cause motor dysfunction (claudication, severe osteoarthrosis, rheumatoid arthritis, gouty arthritis, etc)
- Unable to complete daily activities independently due to various diseases and physical weakness before this disease, which seriously affects the effectiveness evaluation
- Known complications affecting drug evaluation, including severe cardiac insufficiency, renal insufficiency, severe mental illness, depression after stroke, dementia, cerebral hemorrhage after cerebral infarction, and so forth
- Known contraindications of Dazhu hongjingtian injection: pregnancy, allergic constitution, multiple drug allergies, allergy to ingredients in Dazhu hongjingtian injection, known adverse reactions to any component of the Dazhu hongjingtian injection
- Controls who used other dosage forms of Dazhu hongjingtian injection (tablets, capsules, etc)
- Unsuitability for participation as judged by the investigators

### Treatment Regimens

All patients will receive current guideline-recommended treatments according to *Chinese Guidelines for the Diagnosis and Treatment of Acute Ischemic Stroke 2018* [15]. The most commonly used medications and treatments include intravenous thrombolysis drugs, antiplatelets, anticoagulants, drugs for volume expansion, neuroprotectants, medicines for improving cerebral blood flow, and traditional Chinese medicine. Cases will be recruited from patients who receive DZHJTI according to the physician’s order. The DZHJTI will be manufactured and supplied by Tonghua Yusheng Pharmaceutical Co, Ltd (approvals Z20060361 and Z20060362), with identical appearance, color, and flavor. The fingerprint-based quality control and ingredient identification met the Chinese pharmacopeia’s reference requirements (detailed information is provided in Multimedia Appendix 1). The administration and dosage of DZHJTI will be determined by the attending physicians based on the patients’ medical history and clinical manifestations.

### Data Collection and Monitoring

Trained research personnel at each participating site will collect baseline data during patient admission, including prehospital care and treatment, prestroke symptoms, and the NIHSS. They will also conduct assessments using the prestroke modified Rankin Scale scores and Barthel Index. Patient information, including demographics, medical and family history, physical examination, auxiliary examination, risk factor assessment, and clinical diagnosis, will be obtained from medical records. The classification of stroke etiology, related complications, and new cerebrovascular events will be documented, and the etiology of AIS will be determined according to the Trial of Org 10172 in Acute Stroke Treatment criteria [17]. Besides standard therapies, the administration of DJHJTI during hospitalization will be documented by the research team.

The collection and reporting of data will use a secure electronic data capture (EDC) system that follows the current clinical protocol and provides each patient with a unique ID. Authorized research personnel can access the EDC system both on personal computers and smartphones, enabling them to complete data collection during daily ward rounds. An overview of the measurement data collection schedule is shown in Table 1. In the EDC system, personal identifiers such as names, ID numbers, and contact information are removed or replaced with unique codes to ensure participant anonymity.

**Table 1:** Schedule of assessments in the cohort study.

Participant assessment	Time point and form				
	Baseline (acute phase)		Follow-up 1	Follow-up 2	Follow-up 3
	Admission	Discharge	1 month	3 months	6 months
	Visit	Visit	Visit or telephone interview	Visit or telephone interview	Visit or telephone interview
Eligibility screen	✓^a^				
Informed consent	✓				
Demographic and anthropometric data	✓				
TCM^b^ questionnaire	✓				
NIHSS^c^	✓	✓			
mRS^d^	✓	✓	✓	✓	✓
BI^e^			✓	✓	✓
Laboratory examination	✓	✓	+^f^	+	+
Imaging examination	✓	✓	+	+	+
Admission status		✓			
Discharge status		✓			
Follow-up interview			✓	✓	✓
AE^g^		+	+	+	+
Outcomes		+	+	+	+

^a^Required data.

^b^TCM: traditional Chinese medicine.

^c^NIHSS: National Institutes of Health Stroke Scale.

^d^mRS: modified Rankin Scale.

^e^BI: Barthel Index.

^f^Optional data to be collected based on the actual occurrence of related events in patients.

^g^AE: adverse events.

### Follow-Up Procedures

All patients will be identified and followed up using individual identity cards, which are mandatory for all Chinese citizens, enabling unambiguous linkages. Research personnel will conduct regular face-to-face or telephone follow-up at 1, 3, and 6 months after stroke onset. Information on patient outcomes, including mortality, functional status, activities of daily living, cognitive function, and cardiovascular or cerebrovascular events, will be obtained during follow-up. Mortality will be confirmed through a death certificate issued by the local civil registry or the attending hospital. New cardiovascular or cerebrovascular events requiring rehospitalization will be verified by the discharge diagnosis. In the absence of hospitalization, a committee responsible for determining end points will evaluate suspected recurrent cardiovascular or cerebrovascular events [18].

### Outcomes

In this study, the primary outcome will be the distribution of scores on the NIHSS before and after treatment among patients. Secondary outcomes will be assessed at different follow-up visits and will include death, a composite of new clinical vascular events, neurological function, and improvement of traditional Chinese medicine syndrome. Safety assessment will be performed by monitoring adverse events. The complete study outcomes are illustrated in Table 2.

**Table 2:** Outcome variables and definitions.

Outcome variables		Definitions
**Primary outcome**		
	Improvement degree of clinical NIHSS^a^ scores	Basic recovery (NIHSS score decreased by more than 90%) Significant improvement (NIHSS score decreased by 46%-89%) Improvement (NIHSS score decreased by 18%-45%) No change (NIHSS score decreased 0%-18% or increased<18%) Deterioration (NIHSS score increased more than 18%) The basic recovery, significant improvement, and improvement were regarded as effective. The no change, deterioration, and death were regarded as inefficient [19]
**Secondary outcomes**		
	Overall mortality	—^b^
	Stroke recurrence	—
	New-onset major vascular events	Major adverse vascular events include ischemic stroke, hemorrhagic stroke, transient ischemic attack, myocardial infarction, and vascular-related death
	Global disability	Measured by BI^c^ and mRS^d^ scale
	improvement of TCM^e^ syndrome	Measured by TCM questionnaire
	Adverse events	Undesirable experiences occurring to participants during the trial are considered to be related to the DJHJTIf

^a^NIHSS: National Institute of Health Stroke Scale.

^b^Not available.

^c^BI: Barthel Index.

^d^mRS: modified Rankin Scale.

^e^TCM: traditional Chinese medicine.

^f^DZHJTI: Dazhu hongjingtian injection.

### Study Sites and Data Source

The cohort study’s steering committee will conduct a rigorous screening process of hospitals nationwide, aiming to represent the population from each region in mainland China. Only hospitals admitting over 100 patients with stroke annually, experienced in multicenter studies, and equipped with magnetic resonance imaging or computed tomography machines will be considered. After a thorough evaluation, 30 participating sites with proven research capabilities and a demonstrated commitment to the study will be selected.

### Quality Control and Management

Before the study begins, the steering committee will create training materials outlining the study protocol and standard operating procedures for patient screening, recruitment, informed consent, and data collection. Research personnel must complete the training videos and pass an examination to obtain a study permit. The end-point judgment committee will establish standard operating procedures for the proper use of scales, outcome assessment, and reporting procedures. An independent data safety and monitoring board will oversee data monitoring to guarantee adherence to the study documentation, reporting procedures, and the study protocol. This board will be responsible for ensuring that the study is conducted in accordance with good clinical practice guidelines.

### Sample Size

According to the previous studies, the ineffective rate assessed by NIHSS score (percentage of patients experiencing no change and deterioration) in controls was 8.75% (7/80), and the ratio of cases to controls was 0.31 [14]. To test the hypothesis that adjuvant treatment with DZHJTI significantly reduces the ineffective rate with a 2-sided α of .05, power of 0.80, and a 20% (n=248) dropout rate, a sample size of 619 cases in each group is required. Nevertheless, a sufficiently large sample size is also necessary to reduce the bias in real-world studies. Therefore, a total of 1000 case participants per group will be included.

### Statistical Analysis

A statistical analysis plan was developed prior to initiating the cohort study. Continuous data will be summarized using mean, median, standard deviation, or IQR, while categorical data will be presented as counts and percentages. Between-group comparisons will be conducted using appropriate parametric tests (such as the 2-tailed Student *t* test) or nonparametric tests (such as the chi-square test, Fisher exact test, or Mann-Whitney *U* test). Time-to-event analysis will involve censoring patients at their last follow-up assessment when experiencing a clinical event, at the end of the study, or at the time of withdrawal from the study. Cumulative clinical events will be reported as Kaplan-Meier estimates. Hazard ratios will be calculated at 95% CIs using Cox proportional hazard regression methods.

During data analysis, both confounding factors and missing values will be taken into account. The augmented inverse propensity weighted (AIPW) estimator will be used to obtain a marginal estimate of the treatment effect rather than a conditional estimate. AIPW analysis creates a pseudopopulation in which the relationship between the confounders and the exposure is blocked. Multiple imputation will be used to handle missing values of covariates, and both complete case and imputed data sets will be analyzed. For each imputed data set, the AIPW estimator will be used to estimate the treatment effect, and Rubin rules will be used to combine these estimates and obtain an overall estimate [20]. Sensitivity analyses for unmeasured confounding in observational studies will be performed using the methodology proposed by VanderWeele and Ding [21]. All statistical tests will be 2-sided with statistical significance set at *P*<.05. Statistical analyses will be conducted using SAS software (version 9.4; SAS Institute Inc).

### Ethical Considerations

This clinical trial will be conducted in accordance with the principles outlined in the Declaration of Helsinki. Prior to enrollment, all participants or their authorized representatives will be provided with a written informed consent form and will be required to provide their consent. The ethics committee of the Institute of Basic Clinical Research, China Academy of Chinese Medical Sciences (P22004/PJ04) granted approval for the trial on July 18, 2022. The authors declare that the results will be submitted to an international peer-reviewed journal for their prompt dissemination.

## Results

The clinical trial registration for this study was approved in October 2022, and recruitment and enrollment of participants commenced in November 2022. We anticipate that the future outcomes of this study will be published in reputable, peer-reviewed health-related research journals around 2025.

## Discussion

We present the rationale and design of a prospective, multicenter observational study that aims to evaluate the clinical effectiveness and safety of DZHJTI in the treatment of AIS in real-world settings. Rhodiola has long been used in Eurasian traditional medicine as a natural tonic, but DZHJTI’s efficiency in treating AIS is not well known because previous studies had limited external validity [22,23]. Thus, it is necessary to collect large longitudinal data to confirm the practical benefits and disadvantages of DZHJTI treatment in patients with stroke. These data can then be used to develop initiatives to improve evidence-based care and patient outcomes.

Adjuvant treatment with DZHJTI has been shown to have an additional positive effect on improving clinical symptoms and hemodynamic indexes in patients with AIS [14,24]. This beneficial effect may be attributed to the ability of its ingredients, including salidroside, tyrosol, and *Rhodiola* polysaccharide, and the fact that it contains flavonoids, phenolic compounds, trace elements, and amino acids. This has been demonstrated to activate antioxidant enzymes’ activity in brain tissue, reduce the oxidative damage of free radicals to cells, and improve glucose and lipid metabolism [25,26]. However, the evidence for the effectiveness of DZHJTI is still limited due to a lack of large controlled studies, and the strict treatment regimes in randomized controlled trials may be not applicable in clinical practice [14]. Therefore, well-designed prospective real-world studies with large sample sizes are necessary to evaluate the evidence.

Our study uses an observational design, and the treatments will not be specified by the study protocol. This approach has the advantage of reflecting real-world clinical practice, including therapy switches and concomitant medications, which can provide valuable insights into treatment effectiveness in diverse patient populations. Additionally, studying how DZHJTI is prescribed by health care providers and used in everyday environments can offer valuable information about the people and settings that are often underrepresented in randomized controlled trials, potentially leading to improvements in the way medicines are prescribed and used among these patient populations.

However, it is important to note that biases are common in real-world studies. Participants from different study sites may differ in characteristics, and combined treatments can vary widely. Additionally, incomplete data can be a significant issue with real-world data sources. Therefore, we plan to use the AIPW estimator, which involves a doubly robust procedure to minimize bias if either the treatment or outcome model is correctly specified [27]. The accuracy of the AIPW estimator for the average treatment effect has been demonstrated under different scenarios of misspecification [27]. To perform propensity score analysis when data are missing, we will conduct the analysis in each imputed data set and then combine the estimates of the treatment effect to obtain an overall estimate. This method has been demonstrated to be unbiased under a missing at random mechanism, and Rubin rules have been shown to provide reliable variance estimates and show good balancing properties [20].

Another key consideration in this trial is selecting an appropriate primary outcome, which depends largely on the disease and should reflect the treatment effect and expected mechanism. NIHSS is a tool used to objectively quantify neurological impairment after stroke [28], while the modified Rankin Scale and modified Barthel Index are commonly used scales to measure disability or dependence in daily activities in patients with stroke or other neurological dysfunction [29]. Previous studies on herbal injection often have limited follow-up periods of up to 3 months due to drug metabolism. However, since AIS neurological function recovery is a slow process, with the main recovery period occurring within 2 to 6 months after onset [30], we evaluated the effects of DZHJTI on neurological recovery for a period of 6 months after discharge from the hospital.

To our knowledge, this is the first and largest prospective cohort study in China that evaluates the real-world effectiveness and safety of DZHJTI in the treatment of AIS. It may provide additional clinical evidence, including the duration of response, long-term drug effectiveness, and subgroup efficacy data of DZHJTI treatment for patients with AIS under the influence of other confounding factors. Ultimately, the results of this study will help optimize individualized interventions with DZHJTI for the treatment of AIS in China, leading to better patient outcomes and more standardized treatment practices.

## Appendix

Multimedia Appendix 1 Fingerprint chromatogram.

## Abbreviations

AIPW: augmented inverse propensity weighted
AIS: acute ischemic stroke
DZHJT: Dazhu hongjingtian
DZHJTI: Dazhu hongjingtian injection
EDC: electronic data capture
ITMCTR: International Traditional Medicine Clinical Trial Registry
NIHSS: National Institute of Health Stroke Scale

## Data Availability

The data sets generated and analyzed during this study are available from the corresponding author on reasonable request.
