# Supplementary material for: Dazhu Hongjingtian Injection for Ischemic Stroke: Protocol for a Prospective, Multicenter Observational Study
Source: JMIR Res Protoc. 2023 Dec 22;12:e52447. doi: 10.2196/52447 (PMC10770785; doi:10.2196/52447)
Supplement: Multimedia Appendix 1 [file resprot_v12i1e52447_app1.docx]

Additional file 1

# Overview

The injections of DZHJT utilized in this study are products manufactured by Tonghua Yusheng Pharmaceutical Company, Limited, following the standard YBZ11852006-2018Z. The Food and Drug Administration of China has granted approval for these injections to be used in the treatment of stable angina and coronary heart disease (Ran et al., 2019).

DZHJT, scientifically known as *Rhodiola Kirilowii* (Regel) Regel, is a member of the family *Crassulaceae* and the genus *Rhodiola* (Zhuang et al., 2019). Roughly 90% of *Rhodiola* species, inclusive of DZHJT, are predominantly found in the northwest, southwest, and northeast regions of China. These species thrive in environments like slopes and rocks, flourishing at altitudes that range from 1600 to 3900 meters (Zhuang et al., 2019).

Salidroside is identified as one of the most potent constituents present in the DZHJT injection. Every milliliter of this product contains no less than 3.5mg of salidroside (C_14_H_20_O_7_) and no less than 0.30mg of leucine (C_8_H_10_O_2_) per 1ml (National Medical Products Administration, 2022).

# Procedure

Take 1670g of DZHJT, add water and decoct three times, each time with 6 times the amount of water, each time for 2 hours, combine the decoction, filter it, concentrate the filtrate to a clear paste with a relative density of 1.15-1.20 (50℃), add ethanol to make the alcohol content reach 70%, stir evenly, refrigerate for 24 hours (5-10℃), filter, wash the precipitate with a small amount of 70% ethanol (5-10℃) once, filter, combine the filtrate, recover the ethanol and concentrate to a clear paste with a relative density of 1.15-1.20 (50℃), add more ethanol to make the alcohol content reach 85%, stir evenly, refrigerate for 24 hours (5-10℃), filter, wash the precipitate with a small amount of 85% ethanol (5-10℃) once, filter, combine the filtrate, adjust the pH to 8.0, refrigerate for 12 hours (5-10℃), filter, recover the ethanol from the filtrate and concentrate to a clear paste with a relative density of 1.20-1.30 (50℃), add 1000ml of injection water, refrigerate for 12 hours (5-10℃), filter, adjust the pH of the filtrate to 7.0, add activated carbon, heat and boil for 30 minutes, refrigerate for 72 hours (5-10℃), remove the carbon and filter, ultrafilter the filtrate with a column capable of retaining molecules with a molecular weight of 10000, concentrate the ultrafiltrate, freeze-dry, add injection water to the freeze-dried substance to make 1000ml, filter through a microporous filter membrane, seal, sterilize, and obtain the product. The drug to solvent ratio is approximately 1:6 and resulting drug to extract ratio is 1.67.

# Characteristics

This product is a clear liquid from light yellow to brown-yellow.

# Identification

Take 10ml of this product, place it in a separatory funnel, shake it with water-saturated n-butanol for extraction 3 times, each time 10ml. Combine the n-butanol liquid, recover the solvent until dry, and the residue is dissolved in 10ml of methanol, which is used as the test solution. Take salidroside and tyrosol references and add methanol to make a mixed solution containing 1mg per 1ml, which is used as the reference solution. According to the Thin Layer Chromatography, take 2-3μl of the above-mentioned reference solution, and 2-10μl of the test solution, and spot them on the same silica gel G thin-layer plate, using trichloromethane-methanol (6:1) as the developing agent. Develop, take out, dry, spray with a mixed solution of 1% potassium ferricyanide-1% ferric chloride (1:1). In the chromatogram of the test product, at the position corresponding to the reference chromatogram, a spot of the same color appears.

# Fingerprint Chromatogram

## Determination by High Performance Liquid Chromatography (National Medical Products Administration, 2022)

**Chromatographic conditions and system suitability test:** Octadecyl silane bonded silica gel is used as the filler (Agilent XDB C_18_ chromatographic column, 150mmx4.6mm, 5μm), with acetonitrile as the mobile phase A and 0.07% phosphoric acid solution as the mobile phase B, and gradient elution is performed according to the table below; the column temperature is 30°C, and the flow rate is 0.8ml per minute; the detection wavelength is 278nm. The theoretical plate number calculated based on the salidroside peak should not be less than 30000.

| Time (min) | Mobile Phase A (%) | Mobile Phase B (%) |
| --- | --- | --- |
| 0~10 | 0 | 100 |
| 10~30 | 0→3 | 100→97 |
| 30~38 | 3→5 | 97→95 |
| 38~45 | 5→7 | 95→93 |
| 45~50 | 7→13 | 93→87 |
| 50~60 | 13→22 | 87→78 |
| 60~70 | 22 | 78 |

**Preparation of reference solution:** Take an appropriate amount of salidroside reference substance, weigh accurately, and prepare a solution containing 0.05mg per 1ml with water.

**Preparation of test solution:** Accurately measure 0.5ml of this product, place it in a 10ml volumetric flask, dilute to the mark with water, shake well, filter, and take the filtrate.

**Measurement method:** Accurately take 20μl each of the reference solution and the test solution, inject them into the liquid chromatograph, and measure. Record the chromatographic peaks within 60 minutes. The chromatographic peak with the same retention time as the reference chromatographic peak should appear in the fingerprint chromatogram of this product. According to the Chinese medicine chromatographic fingerprint similarity evaluation system, the similarity between the whole chromatogram of the test product fingerprint and the reference fingerprint chromatogram should not be less than 0.90.
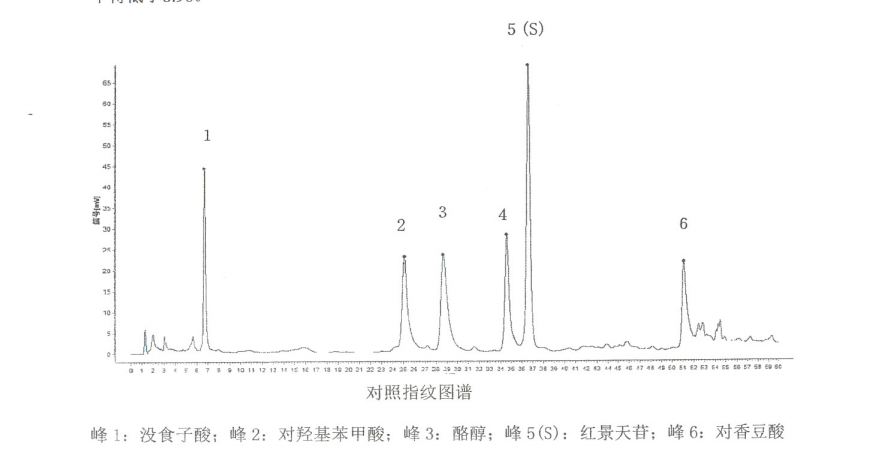


**Figure:** Fingerprint diagram for the extraction of Dazhuhongjingtian. Peak 1: Gallic acid; Peak 2: p-Hydroxybenzoic acid; Peak 3: Tyrosol; Peak 5(S): salidroside; Peak 6: p-Coumaric acid

## Determination by Ultra-high performance liquid chromatography (Du ranran et al., 2017)

**Instruments:** Ultra-high performance liquid chromatography (Waters Acquity UPLC), Acquity UPLC BEH Shield RP18 (2.1× 100 mm, 1.7 µm) chromatographic column, both from Waters Corporation, USA. BT125D one hundred thousandth balance from Sartorius Scientific Instruments Co., Ltd., Germany.

**Reagents:** DZHJT injection (Tonghua Yusheng Pharmaceutical Co., Ltd.). Salidroside reference substance (National Institute for Food and Drug Control, content calculated as 99.8 %, batch number: 110818-201206). Chromatographically pure methanol (Sigma, USA), chromatographically pure formic acid (Tianjin Kemiou Chemical Reagent Co., Ltd.), water is ultrapure water.

**Chromatographic analysis:** The chromatographic column is Acquity UPLC BEH Shield RP18 (2.1× 100 mm, 1.7 µm); the mobile phase is 0.1% formic acid water(A)-methanol(B); sample temperature: 25° C, column temperature: 40° C; flow rate: 0.2mL/min; injection volume: 3 μ L, detection wavelength 275 nm. The elution program is 0~13 min: 5% B~18% B.

**Preparation of reference solution and test solution:** Reference solution: Accurately weigh an appropriate amount of salidroside reference substance, place it in a 25 mL brown volumetric flask, dilute to the mark with distilled water, and obtain the reference reserve solution.

**Test solution:** Accurately measure 1mL of DZHJT injection into a 25 mL brown volumetric flask, add distilled water to the mark, shake well, and let stand at room temperature. After filtering through a 0.22 μ m microporous membrane, sample the UPLC chromatographic analysis under the chromatographic conditions in Chromatographic condition analysis. The obtained chromatogram is shown in figure below, and salidroside is well separated.


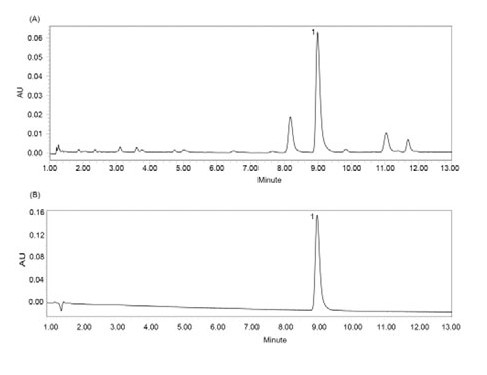


**Figure**: UPLC Chromatogram (275 nm) (A) DZHJT Injection Sample; (B) Reference Substance (1- Salidroside)

## Determination by High Performance Liquid Chromatography (Zhi Xuran et al., 2019)

### Instruments and Reagents

The Waters 2695 High Performance Liquid Chromatograph, equipped with a 2487 dual-channel ultraviolet-visible detector (Waters, USA); AB204-S model balance with a precision of one ten-thousandth (Mettler Toledo, Switzerland); GWF-8JD particle analyzer (Tianjin Tianhe Analytical Instrument Co., Ltd.); FE20 Laboratory pH meter (Mettler Toledo Instruments Co., Ltd.); 102CR Electric blast drying oven (Shandong Weifang Jingying Medical Equipment Company).

Methanol is chromatographically pure [Thermo Fisher Scientific (China) Co., Ltd.]; Phosphoric acid is analytically pure (Shijiazhuang Yellow Phosphorus Factory); Water is purified water (Hangzhou Wahaha Group Co., Ltd.). Reference substances: salidroside (Batch number: HJTG2016012001), tyrosol (Batch number: LC2015121601) were purchased from Nanjing Spring and Autumn Bioengineering Co., Ltd., both with purity >98%; 5% Glucose injection (China Resources Double-Crane Pharmaceutical Co., Ltd., Batch number: H201611191; Specification: 250 mL).

### Methods and Results

**Preparation of combined solution:** According to the instructions of DZHJT injection and the commonly used dosage in clinical practice, the solution was prepared in the intravenous drug dispensing center's clean bench: 10 mL (2 vials) of DZHJT injection was diluted with 250 mL of 5% glucose injection. The prepared solution was divided into 3 groups, each with 3 portions, placed in different environments such as room temperature (normal light, 10~30 ℃), light exposure (4500 Lx lamp irradiation in black box), and constant temperature of 40 ℃, to determine the insoluble particles, pH value, and effective component (salidroside and tyrosol) content at different time points 0, 2, 4, 8, 12, 24 h.

**Preparation of reference solution:** Weigh an appropriate amount of salidroside and tyrosol reference substances and add an appropriate amount of methanol to make a reference reserve solution (concentration of salidroside and tyrosol is 1.800 and 1.360 mg·mL^-1^ respectively).

Take an appropriate amount of the prepared salidroside and tyrosol reserve solution, add methanol to prepare a mixed reference solution (concentration of salidroside and tyrosol is 270.0 and 136.0 μg·mL^-1^ respectively), vortex for 2 min, and set aside.

**Preparation of test solution:** Filter the prepared DZHJT injection with a 0.22 µm pore filter, take the clarified filtrate as the test solution.

**Chromatographic conditions:** Use Symmetry® C18 chromatographic column (4.6 mm×250 mm, 5 μm), mobile phase: methanol (chromatographically pure)-0.4% phosphoric acid aqueous solution (25:75). Flow rate: 1.0 mL·min^-1^; Detection wavelength: 275 nm. Column temperature: 30 ℃, injection volume: 10 μL. The chromatograms of blank solution, reference solution (concentration of salidroside is 33.75 μg·mL^-1^, tyrosol concentration is 17.00 μg·mL^-1^) and test solution are shown in figure below.


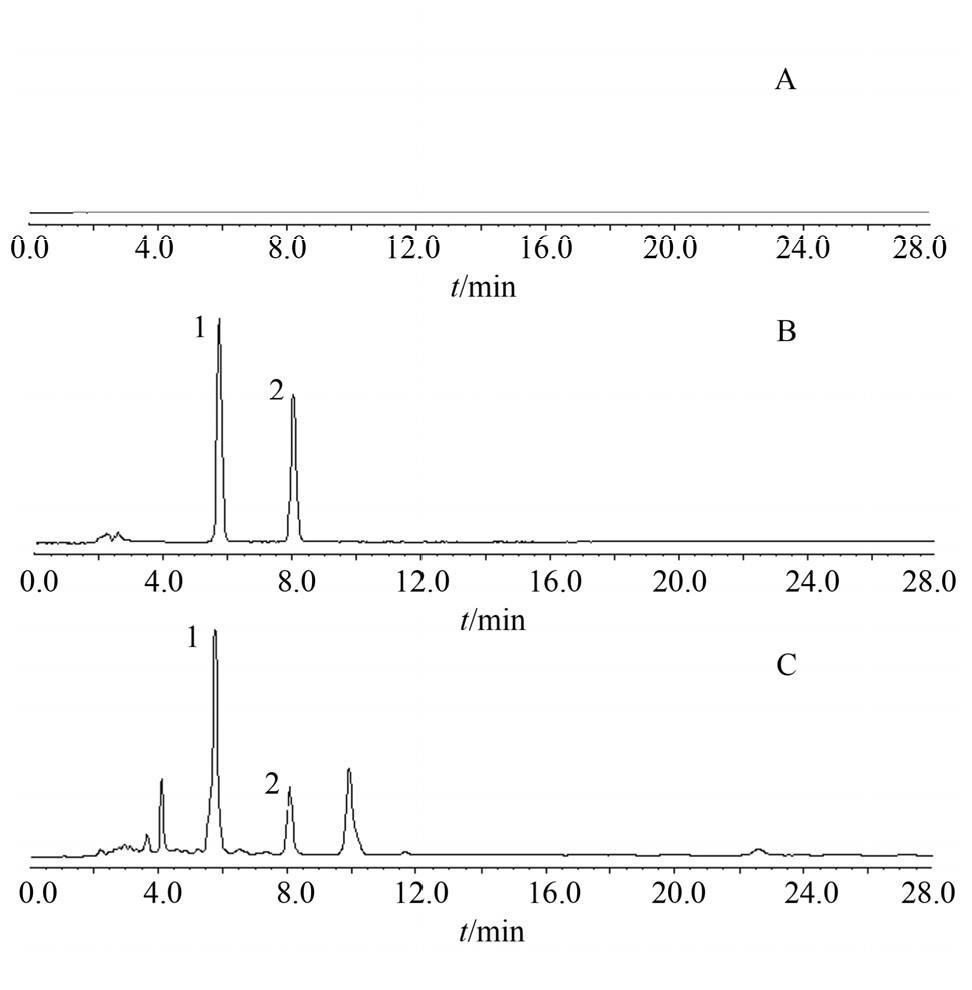


**Figure:** High-performance liquid chromatogram; A: blank control; B: reference standard; C: sample; 1: salidroside; 2: tyrosol.

# Usage and Dosage

Intravenous drip. 10ml at a time, added to 250ml of 5% glucose injection solution, once a day. A course of treatment lasts 10 days or follow the doctor's advice.


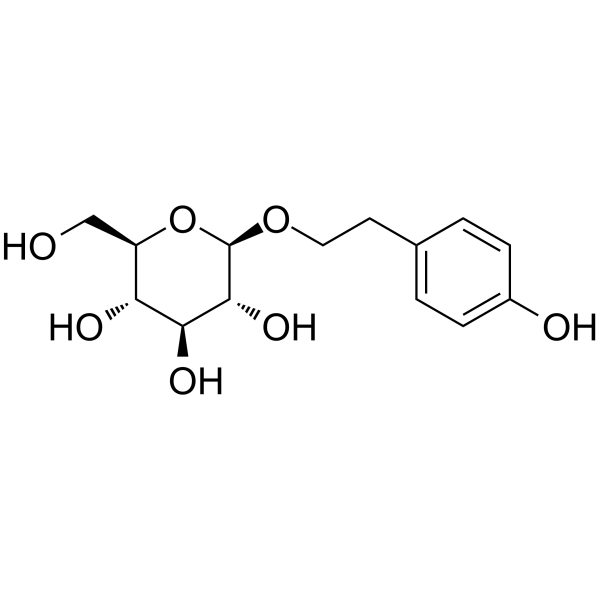


**Figure:** Salidroside molecular Formula C_14_H_20_O_7_


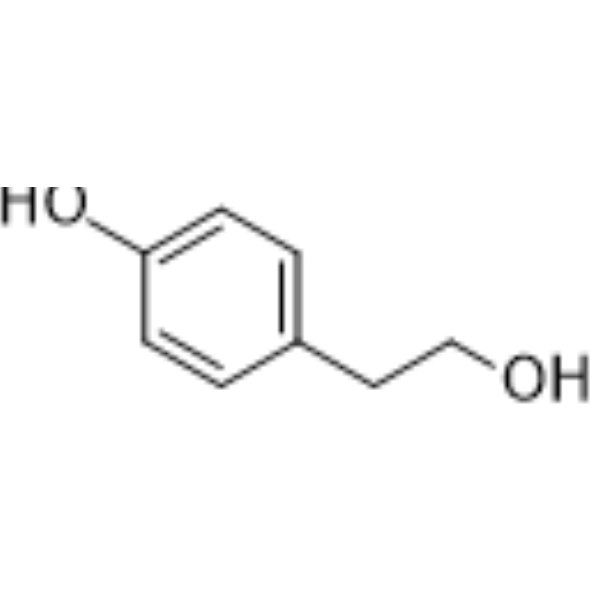


**Figure:** Tyrosol molecular Formula: C_8_H_10_O_2_

# References:

Campeau, L. (1976). Letter: Grading of angina pectoris. *Circulation* 54, 522–523. doi: 10.1161/CIRC.54.3.947585.

National Medical Products Administration (2022). Dazhu Hongjingtian injection [YBZ11852006]. *https://www.nmpa.gov.cn/zwfw/sdxx/ypbzbbjfb/index.html.*

Ran, N., Pang, Z., Guan, X., Wang, G., Liu, J., Li, P., et al. (2019). Therapeutic Effect and Mechanism Study of Rhodiola wallichiana var. cholaensis Injection to Acute Blood Stasis Using Metabolomics Based on UPLC-Q/TOF-MS. *Evidence-based Complementary and Alternative Medicine* 2019. doi: 10.1155/2019/1514845.

Spertus, J. A., Winder, J. A., Dewhurst, T. A., Deyo, R. A., Prodzinski, J., McDonnell, M., et al. (1995). Development and evaluation of the Seattle Angina questionnaire: A new functional status measure for coronary artery disease. *J Am Coll Cardiol* 25, 333–341. doi: 10.1016/0735-1097(94)00397-9.

Du ranran, Li Yue, and Liu Liping (2017). [UPLC method to determine the content of salidroside in large plant Rhodiola rosea injection]. *Inner Mongolia Traditional Chinese Medicine* 36, 1.

Zhi Xuran, Liu Hongtao, Bai Wanjun, Wang Mi, and Dong Zhanjun (2019). [Study on the Compatibility Stability of Sofren Injection]. *Modern Applied Pharmacy in China* 36, 4.

Zhuang, W., Yue, L., Dang, X., Chen, F., Gong, Y., Lin, X., et al. (2019). Rosenroot (Rhodiola): Potential Applications in Aging-related Diseases. *Aging Dis* 10, 134. doi: 10.14336/AD.2018.0511.
